# Supplementary material for: Progression-free survival and overall survival after BRCA1/2-associated epithelial ovarian cancer: A matched cohort study
Source: PLoS One. 2022 Sep 22;17(9):e0275015. doi: 10.1371/journal.pone.0275015 (PMC9498928; doi:10.1371/journal.pone.0275015)
Supplement: S1 Table — (DOCX) [file pone.0275015.s001.docx]

S1 Table. Patient and tumor characteristics – dataset for prospective analyses

|  | ***BRCA1*** |  | **Sporadic** |  |  |  | ***BRCA2*** |  | **Sporadic** |  |  |
| --- | --- | --- | --- | --- | --- | --- | --- | --- | --- | --- | --- |
|  | **N** | **%** | **N** | **%** | **p-value** |  | **N** | **%** | **N** | **%** | **p-value** |
|  | 73 |  | 73 |  |  |  | 9 |  | 9 |  |  |
| **Follow-up, median years (range)** | 4.7 | (0.1-16) | 3.8 | 0.1-16.4 | .327 |  | 4.2 | (1.2-10.1) | 4.7 | (1.7-6.4) | .895 |
| **Year of birth, median (range)** | 1955 | (1925-1976) | 1954 | (1923-1977) | .806 |  | 1952 | (1932-1960) | 1953 | (1938-1956) | .451 |
| **DNA test result** |  |  |  |  |  |  |  |  |  |  |  |
| Median age, median (range) | 48 | (26-75) |  |  |  |  | 58 | (42-70) |  |  |  |
| Time between EOC diagnosis and DNA test result, median years (range) | NA |  |  |  |  |  | NA |  |  |  |  |
| before EOC | 73 | (100%) |  |  |  |  | 9 | (100%) |  |  |  |
| **Year of EOC diagnosis, median (range)** | 2006 | (1997-2015) | 2007 | (1992-2014) | .944 |  | 2008 | (2004-2014) | 2010 | (2000-2014) | .503 |
| **Age at EOC diagnosis, median (range)** | 52 | (32-78) | 52 | (33-76) | .723 |  | 60 | (45-75) | 54 | (47-75) | .757 |
| **FIGO** |  |  |  |  |  |  |  |  |  |  |  |
| Low (≤IIA) | 11 | (19%) | 15 | (24%) | .486 |  | 1 | (13%) | 1 | (13%) | 1 |
| High (≥IIB) | 48 | (81%) | 48 | (76%) |  |  | 7 | (87%) | 7 | (87%) |  |
| unknown | 14 |  | 10 |  |  |  | 1 |  | 1 |  |  |
| **Grade** |  |  |  |  |  |  |  |  |  |  |  |
| Well differentiated | 0 | (0%) | 8 | (15%) | <0.005 |  | 0 | (0%) | 0 | (0%) | NA |
| Poorly differentiated | 58 | (100%) | 46 | (85%) |  |  | 7 | (100%) | 7 | (100%) |  |
| unknown | 15 |  | 19 |  |  |  | 2 |  | 2 |  |  |
| **Histology** |  |  |  |  |  |  |  |  |  |  |  |
| Serous | 61 | (85%) | 39 | (53%) | <0.005 |  | 8 | (89%) | 4 | (45%) | .255 |
| Endometrioid | 2 | (3%) | 11 | (15%) |  |  | 0 | (0%) | 2 | (22%) |  |
| Clear cell | 0 | (0%) | 6 | (8%) |  |  | 0 | (0%) | 0 | (0%) |  |
| Mucinous | 2 | (3%) | 2 | (3%) |  |  | 0 | (0%) | 1 | (11%) |  |
| Adenocarcinoma NOS | 7 | (9%) | 13 | (18%) |  |  | 1 | (11%) | 1 | (11%) |  |
| Other | 0 | (0%) | 2 | (3%) |  |  | 0 | (0%) | 1 | (11%) |  |
| Unknown | 1 |  | 0 |  |  |  | 0 |  | 0 |  |  |
| **CA125 (U/ml)** |  |  |  |  |  |  |  |  |  |  |  |
| ≤35 | 15 | (27%) | 4 | (6%) | <0.01 |  | 0 | (0%) | 0 | (0%) | .429 |
| 35-500 | 20 | (36%) | 25 | (40%) |  |  | 0 | (0%) | 2 | (25%) |  |
| >500 | 20 | (36%) | 33 | (53%) |  |  | 2 | (100%) | 6 | (75%) |  |
| unknown | 18 |  | 11 |  |  |  | 7 |  | 1 |  |  |
| **Type of chemotherapy** |  |  |  |  |  |  |  |  |  |  |  |
| platinum & anthracyclines | 0 | (0%) | 2 | (3%) | .228 |  | 0 | (0%) | 0 | (0%) | .303 |
| platinum & taxanen | 65 | (90%) | 64 | (88%) |  |  | 8 | (89%) | 9 | (100%) |  |
| platinum | 5 | (7%) | 7 | (10%) |  |  | 0 | (0%) | 0 | (0%) |  |
| taxanen & anthracyclines | 0 | (0%) | 0 | (0%) |  |  | 0 | (0%) | 0 | (0%) |  |
| taxanen | 2 | (3%) | 0 | (0%) |  |  | 1 | (11%) | 0 | (0%) |  |
| unknown | 1 |  | 0 |  |  |  | 0 |  | 0 |  |  |
| **Timing of chemotherapy** |  |  |  |  |  |  |  |  |  |  |  |
| Neoadjuvant | 8 | (12%) | 26 | (37%) | <0.005 |  | 1 | (14%) | 2 | (22%) | .687 |
| Adjuvant | 60 | (88%) | 45 | (63%) |  |  | 6 | (86%) | 7 | (78%) |  |
| unknown | 5 |  | 2 |  |  |  | 2 |  | 0 |  |  |
| **Debulking surgery** |  |  |  |  |  |  |  |  |  |  |  |
| No | 6 | (8%) | 7 | (10%) | .731 |  | 0 | (0%) | 0 | (0%) | NA |
| Yes (primary or interval) | 67 | (92%) | 64 | (90%) |  |  | 8 | (100%) | 9 | (100%) |  |
| unknown | 0 |  | 2 |  |  |  | 1 |  | 0 |  |  |
| **Complete debulking** |  |  |  |  |  |  |  |  |  |  |  |
| No | 20 | (48%) | 14 | (36%) | .285 |  | 2 | (67%) | 1 | (20%) | .187 |
| Yes | 22 | (52%) | 25 | (64%) |  |  | 1 | (33%) | 4 | (80%) |  |
| Unknown | 25 |  | 25 |  |  |  | 5 |  | 4 |  |  |
| **Recurrent disease** | 47 | (64%) | 55 | (75%) | .149 |  | 5 | (56%) | 6 | (67%) | .629 |
| Age at 1^st^ recurrence, median (range) | 54 | (33-79) | 54 | (35-77) | .775 |  | 63 | (56-70) | 56 | (48-63) | .068 |
| Year of 1^st^ recurrence, median (range) | 2007 | (1999-2017) | 2009 | (1993-2019) | .245 |  | 2009 | (2007-2014) | 2010 | (2001-2016) | .914 |
| Time between diagnosis of EOC and 1^st^ recurrence, median months (range) | 19.6 | (0.6-143.7) | 15.1 | (0.6-92.1) | .093 |  | 23.3 | (9.2-42.8) | 22.4 | (9.6-34.1) | .855 |
| Chemotherapy after recurrence |  |  |  |  |  |  |  |  |  |  |  |
| No | 7 | (15%) | 12 | (24%) | .259 |  | 1 | (20%) | 1 | (17%) | .887 |
| Yes | 40 | (85%) | 38 | (76%) |  |  | 4 | (80%) | 5 | (83%) |  |
| Unknown | 0 |  | 5 |  |  |  | 0 |  | 0 |  |  |
| PARPi after recurrence |  |  |  |  |  |  |  |  |  |  |  |
| No | 39 | (83%) | 49 | (98%) | <0.05 |  | 3 | (60%) | 5 | (100%) | .114 |
| Yes | 8 | (17%) | 1 | (2%) |  |  | 2 | (40%) | 0 | (0%) |  |
| Unknown | 0 |  | 5 |  |  |  | 0 |  | 1 |  |  |
| **Deceased** | 42 | (58%) | 47 | (64%) | .396 |  | 5 | (56%) | 6 | (67%) | .629 |
| Age at death, median (range) | 56 | (34-83) | 55 | (34-77) | .227 |  | 65 | (59-76) | 59 | (49-67) | .144 |
| Time between 1^st^ recurrence and death, median months (range) | 24 | (0-78.9) | 10.8 | (0-90.6) | .061 |  | 23.7 | (15.7-58.8) | 26.1 | (10.6-51.2) | .807 |
| Time between diagnosis of EOC and death, median months (range) | 48.7 | (0.6-154.7) | 24.9 | (1-170.5) | <0.01 |  | 50.2 | (14-101.6) | 50.6 | (20.2-66.4) | 1 |
| Abbreviations: EOC, epithelial ovarian cancer; PARPi, poly(ADP-ribose) polymerase inhibitors | | | | | | | | | | | |
