# Supplementary material for: Progression-free survival and overall survival after BRCA1/2-associated epithelial ovarian cancer: A matched cohort study
Source: PLoS One. 2022 Sep 22;17(9):e0275015. doi: 10.1371/journal.pone.0275015 (PMC9498928; doi:10.1371/journal.pone.0275015)
Supplement: S2 Table — (DOCX) [file pone.0275015.s002.docx]

S2 Table. Association of *BRCA1* and *BRCA2* pathogenic variant status with progression-free survival and overall survival for the left-truncated analyses

|  | **Progression-free survival** | | | | | **Overall survival** | | | | |
| --- | --- | --- | --- | --- | --- | --- | --- | --- | --- | --- |
|  | N | **PYO** | **Events** | **Rec. rate^1^ (95% CI)** | **HR (95% CI)^2^** | **N** | **PYO** | **Events** | **Mort. rate^1^ (95% CI)** | **HR (95% CI)^2^** |
| ***Left-truncated analyses*** |  |  |  |  |  |  |  |  |  |  |
| **Total observation period** |  |  |  |  |  |  |  |  |  |  |
| *BRCA1* | 272 | 1227 | 190 | 155 (134-179) | 0.69 (0.57-0.84) | 353 | 2303 | 243 | 106 (93-120) | 0.78 (0.65-0.93) |
| sporadic | 272 | 1017 | 215 | 211 (185-242) | 1 | 353 | 1973 | 264 | 134 (119-151) | 1 |
| **Observation period < *t*** | ***t* = 3.2 yrs** | |  |  |  | ***t* = 6 yrs** | |  |  |  |
| *BRCA1* | 272 | 607 | 145 | 239 (203-281) | 0.63 (0.51-0.79) | 353 | 1567 | 178 | 114 (98-132) | 0.65 (0.53-0.79) |
| sporadic | 272 | 492 | 177 | 359 (310-417) | 1 | 353 | 1269 | 229 | 181 (159-205) | 1 |
| **Observation period ≥ *t*** |  |  |  |  |  |  |  |  |  |  |
| *BRCA1* | 114 | 620 | 45 | 73 (54-97) | 1.01 (0.65-1.55) | 144 | 736 | 65 | 88 (69-113) | 1.65 (1.09-2.5) |
| sporadic | 84 | 525 | 38 | 72 (53-100) | 1 | 107 | 704 | 35 | 50 (36-69) | 1 |
| ***Left-truncated analyses*** |  |  |  |  |  |  |  |  |  |  |
| **Total observation period** |  |  |  |  |  |  |  |  |  |  |
| *BRCA2* | 87 | 453 | 54 | 119 (91-156) | 0.5 (0.35-0.72) | 111 | 849 | 69 | 81 (64-103) | 0.56 (0.4-0.78) |
| sporadic | 87 | 308 | 68 | 221 (174-280) | 1 | 111 | 630 | 82 | 130 (105-162) | 1 |
| **Observation period < *t*** | ***t* = 2.9 yrs** | |  |  |  | ***t* = 5.9 yrs** | |  |  |  |
| *BRCA2* | 87 | 204 | 37 | 182 (132-251) | 0.39 (0.26-0.6) | 111 | 528 | 42 | 80 (59-108) | 0.35 (0.24-0.52) |
| sporadic | 87 | 152 | 59 | 389 (302-502) | 1 | 111 | 399 | 77 | 193 (154-241) | 1 |
| **Observation period ≥ *t*** |  |  |  |  |  |  |  |  |  |  |
| *BRCA2* | 47 | 249 | 17 | 68 (42-110) | 1.15 (0.51-2.6)^3^ | 58 | 321 | 27 | 84 (58-123) | 4.14 (1.57-10.97)^3^ |
| sporadic | 26 | 156 | 9 | 58 (30-111) | 1 | 30 | 230 | 5 | 22 (9-52) | 1 |
| Abbreviations: N, number of patients; PYO, person-years of observation; Rec. rate, recurrence rate; Mort. Rate, mortality rate; HR, hazard ratio; 95% CI, 95% confidence interval; *t,* time point where HR switches from under to above 1 (in years of observation after diagnosis of epithelial ovarian cancer)  ^1^ per 1000 PYO  ^2^ adjusted for debulking surgery (yes/no)  ^3^ univariable analysis; adjusting for debulking surgery omitted due to zero patients without debulking surgery | | | | | | | | | | |
